# Multiomics profiling of DNA methylation, microRNA, and mRNA in skeletal muscle from monozygotic twin pairs discordant for type 2 diabetes identifies dysregulated genes controlling metabolism

**DOI:** 10.1186/s12916-024-03789-y

**Published:** 2024-12-02

**Authors:** Charlotte Ling, Magdalena Vavakova, Bilal Ahmad Mir, Johanna Säll, Alexander Perfilyev, Melina Martin, Per-Anders Jansson, Cajsa Davegårdh, Olof Asplund, Ola Hansson, Allan Vaag, Emma Nilsson

**Affiliations:** 1 Epigenetics and Diabetes Unit, Department of Clinical Sciences, Lund University Diabetes Centre, Lund University, Scania University Hospital, Malmö, 205 02 Sweden; 2 Department of Cellular and Molecular Medicine, University of Copenhagen, Copenhagen, Denmark; 3 Genomics, Diabetes and Endocrinology Unit, Department of Clinical Sciences, Lund University Diabetes Center, Lund University, Malmö, Sweden; 4 Wallenberg Laboratory, Department of Molecular and Clinical Medicine, Sahlgrenska University Hospital, Bruna Straket 16, Level 2/3, Gothenburg, 413 45 Sweden; 5 Institute for Molecular Medicine Finland (FIMM), Helsinki University, Helsinki, Finland; 6 Steno Diabetes Center Copenhagen, Herlev, Denmark; 7 Lund University Diabetes Centre, Lund University, Malmö, 205 02 Sweden; 8 Department of Endocrinology, Scania University Hospital, Malmö, 205 02 Sweden

**Keywords:** Type 2 diabetes (T2D), Skeletal muscle, DNA methylation, Epigenetics, Gene expression, MicroRNA (miRNA), Discordant monozygotic twins

## Abstract

**Background:**

A large proportion of skeletal muscle insulin resistance in type 2 diabetes (T2D) is caused by environmental factors.

**Methods:**

By applying multiomics mRNA, microRNA (miRNA), and DNA methylation platforms in biopsies from 20 monozygotic twin pairs discordant for T2D, we aimed to delineate the epigenetic and transcriptional machinery underlying non-genetic muscle insulin resistance in T2D.

**Results:**

Using gene set enrichment analysis (GSEA), we found decreased mRNA expression of genes involved in extracellular matrix organization, branched-chain amino acid catabolism, metabolism of vitamins and cofactors, lipid metabolism, muscle contraction, signaling by receptor tyrosine kinases pathways, and translocation of glucose transporter 4 (GLUT4) to the plasma membrane in muscle from twins with T2D. Differential expression levels of one or more predicted target relevant miRNA(s) were identified for approximately 35% of the dysregulated GSEA pathways. These include miRNAs with a significant overrepresentation of targets involved in GLUT4 translocation (miR-4643 and miR-548z), signaling by receptor tyrosine kinases pathways (miR-607), and muscle contraction (miR-4658). Acquired DNA methylation changes in skeletal muscle were quantitatively small in twins with T2D compared with the co-twins without T2D. Key methylation and expression results were validated in muscle, myotubes, and/or myoblasts from unrelated subjects with T2D and controls. Finally, mimicking T2D-associated changes by overexpressing miR-548 and miR-607 in cultured myotubes decreased expression of target genes, *GLUT4* and *FGFR4*, respectively, and impaired insulin-stimulated phosphorylation of Akt (Ser473) and TBC1D4.

**Conclusions:**

Together, we show that T2D is associated with non- and epigenetically determined differential transcriptional regulation of pathways regulating skeletal muscle metabolism and contraction.

**Supplementary Information:**

The online version contains supplementary material available at 10.1186/s12916-024-03789-y.

## Background

The prevalence of type 2 diabetes (T2D) is rapidly increasing worldwide. Combinations of genetic and non-genetic factors such as an impaired intrauterine environment, obesity, physical inactivity, and aging increase the susceptibility of this complex metabolic disorder characterized by chronic hyperglycemia. Skeletal muscle is the primary tissue responsible for a major proportion of insulin-stimulated glucose uptake and this function is impaired in T2D [1, 2] and in first-degree relatives without diabetes [3, 4]. However, the exact molecular mechanisms behind the development of skeletal muscle dysfunction in subjects with T2D are not yet fully understood.

A few studies have previously investigated the mRNA expression pattern in skeletal muscle in relation to T2D [5–7] and insulin resistance [8]. These studies found, e.g., downregulation of genes related to glucose uptake, oxidative phosphorylation, and insulin signaling in subjects with T2D compared with controls, and genes involved in lipid metabolism, autophagy, and mTOR signaling associated with skeletal muscle insulin resistance. However, how the differential gene expression itself is regulated in T2D is less well known and may include epigenetic factors such as alterations in DNA methylation and microRNA (miRNA) patterns [9]. Indeed, we have previously found differential miRNA changes and subtle DNA methylation differences in skeletal muscle from a smaller subset of monozygotic (MZ) twin pairs discordant for T2D [10, 11].

**Fig. 1:**
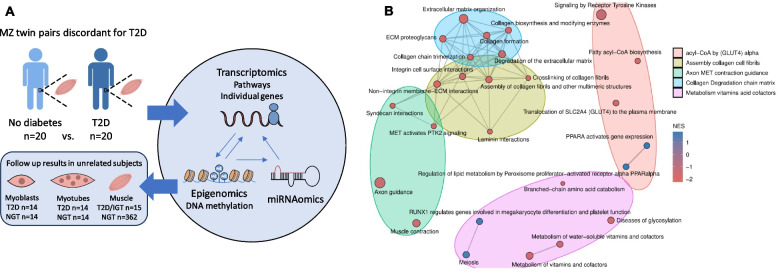
Schematic overview of the project and gene sets with differential expression in skeletal muscle of twins with T2D compared with co-twins without T2D (gene set enrichment analysis with *q* < 0.1). **A** Skeletal muscle biopsies from 20 MZ twin pairs discordant for type 2 diabetes (T2D) were investigated using multiomics, at the mRNA expression, DNA methylation, and miRNA expression levels using arrays. We further used available mRNA expression array data from skeletal muscle (15 subjects with T2D or impaired glucose tolerance (IGT) and 362 unrelated subjects with normal glucose tolerance (NGT)), and mRNA expression and DNA methylation data from myotubes and myoblasts (14 subjects with T2D and 14 unrelated subjects with NGT) to follow up and validate our findings in the twins. **B** An emapplot visualizing the results of the gene set enrichment analysis of the transcriptome data generated in the MZ twin pairs discordant for T2D presented in Table 2 and Supplemental Table 2. The ReactomePA package from Bioconductor (https://doi.org/10.1039/c5mb00663e) was used to generate the emapplot. NES, normalized enrichment scores

Our aim in the present study was to further dissect the molecular mechanisms underlying T2D by using a combination of genome-wide mRNA expression, miRNA expression, and DNA methylation data in skeletal muscle from 20 MZ twin pairs discordant for T2D (Fig. 1). The study of discordant MZ twin pairs offers the opportunity to control for several potential confounders, such as differences in genetic background, early-life environmental exposure, age, and sex. We also used mRNA expression array data from skeletal muscle [12, 13] and mRNA expression and DNA methylation data of myotubes and myoblasts from unrelated subjects with T2D and normal glucose tolerance (NGT) [14] to follow up our findings in the MZ twin pairs discordant for T2D and to test if identified alterations can be found already at progenitor stage and be partly validated (Fig. 1A). We finally performed functional validation experiments, mimicking T2D-associated alterations, of selected candidate miRNAs in cultured human myotubes.

## Methods

### Study participants and clinical examination

Twenty MZ twin pairs discordant for T2D recruited through Scandinavian twin registries were included in the study (nine Swedish and eleven Danish twin pairs). Zygosity was confirmed by analysis of 730,525 genetic markers using HumanOmniExpress arrays (Illumina, San Diego, CA, USA). After an overnight fast, vastus lateralis muscle biopsies were obtained under local anesthesia, frozen in liquid nitrogen, and stored at − 80 °C. Clinical characteristics are described in Table 1 and a subset of the cohort has been described in a previous publication [15]. Moreover, skeletal muscle from eleven of the twin pairs have previously been studied using Illumina 27 k arrays covering ~ 27,000 probes [11] and miRCURY locked nucleic acid (LNA) arrays (Exiqon, Vedbaek, Denmark) [10]. Glucose tolerance was measured by a 75 g oral glucose tolerance test, and T2D determined according to the 1999 World Health Organization criteria. The MZ twins with T2D were treated with oral agents (metformin, sulfonylurea, or rosiglitazone) and/or insulin. All study participants gave informed consent. The study was carried out in accordance with the Declaration of Helsinki.

**Table 1:** Clinical characteristics of study subjects in the T2D discordant MZ twin cohort

	**Discordant MZ twins**		
	No diabetes	T2D	*P*
*N* (male/female)	20 (12/8)	20 (12/8)	
Age (years)	66 ± 7	66 ± 7	
BMI (kg/m^2^)	29.2 ± 5.9	31.3 ± 6.2	0.002
Fasting plasma glucose (mmol/l)	6.1 ± 0.6	9.8 ± 2.5	5.5 × 10^−6^
2-h glucose (mmol/l)	8.6 ± 1.7	17.5 ± 4.8	8.9 × 10^−8^
HbA1c (%)	5.8 ± 0.4	7.4 ± 1.6	0.0003
HbA1c (mmol/mol)	40.5 ± 4.6	57.5 ± 17.6	0.0003

Data are shown as the mean ± SD. Among the discordant twins, the co-twins without T2D exhibited normal glucose tolerance (NGT) in 5 pairs and impaired glucose tolerance (IGT) in 15 pairs. Statistics (twins with T2D versus co-twins without T2D) were calculated using a two-tailed paired *t*-test

### RNA and DNA extractions from skeletal muscle biopsies

Total RNA was extracted using the miRNeasy kit (Qiagen, Hilden, Germany). DNA was extracted using the DNeasy Blood and Tissue kit (Qiagen). RNA/DNA quantity and purity were determined spectrophotometrically (NanoDrop Technologies, Wilmington, DE, USA). RNA integrity was determined using the Experion system (Bio-Rad, Hercules, CA, USA).

### mRNA expression arrays

Total RNA was purified by using the RNeasy MinElute Cleanup kit (Qiagen). mRNA expression was analyzed in skeletal muscle from 19 discordant twin pairs with good quality data using GeneChip Human Gene 1.0 ST arrays (Affymetrix, Santa Clara, CA, USA) according to the manufacturer’s recommendations. We computed Robust Multichip Average (RMA) expression measures using the Oligo package in Bioconductor [16]. Comparisons between discordant twins were based on paired two-tailed Wilcoxon statistics. mRNA expression data were also analyzed by gene set enrichment analysis (GSEA) using reactome pathways (fgsea and reactome.db packages, Bioconductor) [17]. All probes on the array corresponding to transcripts with identifiers were used and ranked according to the *t*-statistics in a paired *t*-test. Intra–twin-pair correlations of within–twin-pair differences in MZ twins were analyzed using Spearman statistics. To follow up the results in the MZ twins discordant for T2D, we used available mRNA expression array data, analyzed using Human Genome U133 Plus 2.0 arrays (Affymetrix), from skeletal muscle of 15 subjects with T2D or impaired glucose tolerance (IGT) and 362 NGT controls [12, 13].

### DNA methylation arrays

DNA methylation was analyzed in skeletal muscle of 17 discordant twin pairs with good quality data using Infinium HumanMethylation450 BeadChips (Illumina). This array contains 485,577 probes, which cover 21,231 (99%) RefSeq genes, 482,421 cytosine guanine dinucleotide (CpG) sites, 3091 non-CpG sites, and 65 random single nucleotide polymorphisms (SNPs) [18]. DNA was treated with bisulfite using the EZ DNA Methylation kit (Zymo Research, Orange, CA). Analysis was performed following the Infinium HD Assay Methylation protocol (Illumina). To limit the effect of batch, co-twins were hybridized on the same chip. The bioinformatics analyses were performed as previously described [19]. Probes with a mean detection *P* value of > 0.01, non-CpG probes, Y-chromosome probes, and 65 SNP probes were removed. *M* values were used for statistical analyses and *β* values, ranging from 0 to 100% methylation, were used to report the outcome. Comparisons between discordant twins were based on paired two-tailed Wilcoxon statistics.

### miRNA expression arrays

miRNA expression was analyzed in skeletal muscle from 19 discordant twin pairs with good quality data using miRNA 3.0 arrays (Affymetrix) according to the manufacturer’s recommendations. RMA expression measures were computed using the Oligo package in Bioconductor (release 3.10) [16]. Comparisons between discordant twins were based on paired two-tailed Wilcoxon statistics. miRPathDB 2.0 was used to identify miRNAs regulating specific pathways [20]. Intra–twin-pair correlations of within–twin-pair differences in MZ twins were analyzed using Spearman statistics.

### Myoblasts and myotubes

Primary muscle stem cells (satellite cells) were isolated from vastus lateralis biopsies obtained from 14 individuals with T2D and 14 NGT unrelated subjects (Additional file 1: Supplemental Table 1). A detailed description of the muscle stem cell isolation and culture has been described previously [14, 21]. DNA and RNA were extracted from the cells using the DNeasy Blood and Tissue kit (Qiagen) and Trizol in combination with RNeasy MinElute Cleanup kit (Qiagen), respectively. mRNA expression was analyzed in 13 subjects with T2D and 13 subjects with NGT based on good quality RNA using HumanHT-12 Expression BeadChip (Illumina) as previously described [14]. The assay procedure followed the manufacturer’s recommendations. DNA methylation was analyzed in all 28 subjects using Infinium Human-Methylation450 BeadChip (Illumina). DNA methylation data was analyzed as previously described [14]. Probes with a mean detection *P* value > 0.01 for more than 60% of the samples were filtered out. Data were background-corrected, log2-transformed, quantile-normalized, and batch-corrected using COMBAT [22]. Linear regression analyses and adjustments for age, BMI, and sex were used to compare DNA methylation and mRNA expression array data between individuals with NGT and T2D. mRNA expression data were also analyzed by GSEA using reactome pathways (fgsea and reactome.db packages, Bioconductor) [17]. The DNA methylation and mRNA expression data were described in a previous study, respectively [14], and the data are available in the NCBI Gene Expression Omnibus (GEO) with accession numbers GSE166467 (https://www.ncbi.nlm.nih.gov/geo/query/acc.cgi?acc=GSE166467) [23] and GSE166652 (https://www.ncbi.nlm.nih.gov/geo/query/acc.cgi?acc=GSE166652) [24].

### Human primary myoblast culture and miRNA transfection

Human primary myoblasts (human skeletal muscle-derived cells (skMDCs) #20, #40, and #68) were obtained from Cook MyoSite® (Pittsburgh, PA, USA). The SkMDCs were maintained at 37 °C and 5% CO_2_ in growth medium (Ham’s F-10 Nutrient Mix, GlutaMAX™ Supplement [#41550021, Gibco®], 20% fetal bovine serum [FBS] [F7524, Sigma], Antibiotic/Antimycotic Solution [#15240062, Gibco®]). Cell culture plates were coated with Matrigel (Standard LDEV-Free Formulation, Corning®) diluted in 1:100 in Dulbecco’s modified eagle medium F12 (DMEM/F12, in-house) and incubated at 37 °C for 30 min. Residual media was aspirated immediately prior to use. When the myoblasts were approximately 90% confluent, growth media was replaced with differentiation media containing DMEM (low glucose, GlutaMAX™ Supplement, pyruvate, No HEPES [#21885025, Gibco®]) supplemented with 2% HS and 100 IU/mL of penicillin–streptomycin (PAN-Biotech, Aidenbach, Germany) for differentiation into myotubes.

Human miR-548z (assay ID: MC19684) and hsa-miR-607 (assay ID: MC11423) mimics (50 nM) and a scrambled negative control (NC, 50 nM) (Thermo Fisher Scientific) were transfected for 5 h using RNAiMAX (#13778075, Thermo Fisher Scientific) in Opti-MEM reduced serum medium at day 4 of differentiation and collected at day 6 for further analyses. Before collection, myotubes were treated with 100 nM insulin for 30 min.

### RNA extraction from cultured human myotubes and qPCR

All protocols were performed according to the manufacturers’ instructions. Total RNA was isolated from miRNA-transfected human myotubes using QIAzol Lysis Reagent (Qiagen). For miRNA first-strand cDNA synthesis, total RNA (50 ng) was reverse-transcribed using the TaqMan microRNA Reverse Transcription (RT) kit (Applied Biosystems, Waltham, MA, USA). A customized RT primer pool was prepared by pooling all miRNA-specific stem-loop primers, hsa-miR-548z (Assay ID: 465137_mat), hsa-miR-607 (Assay ID: 001570), and U6 snRNA (Assay ID: 001973). Additionally, 250 ng RNA was reverse-transcribed using the QuantiTect Reverse Transcription kit (Qiagen). RNA expression analysis was performed by qPCR on a ViiA 7 Real-Time PCR System (miRNA) or QuantStudio 7 Flex System (mRNA) (both Applied Biosystems). MicroRNA expression levels were measured using specific primer–probe sets and the TaqMan Universal Master Mix II, no UNG (Applied Biosystems). mRNA expression levels were measured using TaqMan Real-Time PCR Assays (FAM reporter dye, *SLC2A4* Assay ID: Hs00168966_m1, and *FGFR4* Assay ID: Hs01106908_m1) and the TaqMan Fast Advanced Master Mix (Applied Biosystems). Target gene CT values were normalized to housekeeping gene CT values and expressed relative to the mean NC using the 2^−ΔΔCT^ method. For miRNA analysis, U6 small nuclear RNA was used as housekeeping gene. For mRNA expression analysis, the geometric mean of *PPIA* (Assay ID: Hs04194521_s1) and *HPRT1* (Assay ID: Hs02800695_m1) was used for normalization. Each sample was assayed in duplicate (miRNA) or triplicate (mRNA) qPCR reactions.

### Western blotting

For analysis of protein phosphorylation, miRNA-transfected human myotubes were washed with ice-cold PBS and lysed on ice using RIPA (50 mM Tris–HCl pH 7.5, 150 mM sodium chloride, 2 mM ethylenediaminetetraacetic acid (EDTA), 1% [v/v] Triton X-100, 0.5% [w/v] sodium deoxycholate, 0.1% [w/v] sodium dodecyl sulfate (SDS)) supplemented with protease (cOmplete, Roche, Basel, Switzerland) and phosphatase (Phosphatase Inhibitor Cocktail I, Abcam, Cambridge, UK) inhibitor cocktails. Homogenates were cleared by centrifugation and protein concentration determined by BCA (Pierce, Waltham, MA, USA). Lysates were boiled in sample buffer (60 mM Tris–HCl pH 6.8, 2% [w/v] SDS, 10% [v/v] glycerol, 2% [v/v] 2-mercaptoethanol) and separated on Criterion TGX Stain-Free 4–15% gels (Bio-Rad, Hercules, CA, USA). Proteins were blotted to LF PVDF membranes (0.45 µm) using the Trans-Blot Turbo Transfer System (Bio-Rad), followed by blocking in 5% [w/v] milk in TBS-T (0.05% [w/v] Tween 20). Blots were incubated with primary antibodies overnight at + 4 °C, followed by incubation with horseradish peroxidase (HRP)-conjugated secondary antibodies for 1 h at room temperature. Blots were visualized by enhanced chemiluminescence in a ChemiDoc MP (Bio-Rad) and signal intensities quantified by ImageLab (version 6.1, Bio-Rad). Phosphorylations were normalized to the total levels of each corresponding protein after stripping and reprobing the membrane, as described previously [14]. To be able to compare samples run on separate gels and blotted to separate membranes, we included a loading reference sample on each gel. The quantified intensities for all samples were then normalized to the loading reference before calculating the relative values. The following antibodies were used (dilution 1:1000): rabbit anti-phospho-Akt Thr308 (RRID: AB_329828), rabbit anti-phospho-Akt Ser473 (RRID: AB_329825), rabbit anti-Akt (RRID: AB_329827), rabbit anti-phospho TBC1D4 (AS160) Thr642 (RRID: AB_2533564), rabbit anti-TBC1D4 (AS160) (RRID: AB_492639), and goat anti-rabbit IgG (HRP) (RRID: AB_2099233).

## Results

### Clinical characteristics

The characteristics of T2D discordant MZ twin pairs included in the study are shown in Table 1. Fasting plasma glucose levels, glucose tolerance, HbA1c levels, and BMI differed significantly between twins with T2D and co-twins without T2D. Among the discordant twins, the co-twins without T2D exhibited NGT in 5 pairs and IGT in 15 pairs. For validation, skeletal muscle from 15 subjects with T2D or IGT and 362 NGT unrelated controls were used [12, 13]. We used a collection of myoblasts and myotubes to examine differential developmental oriented mechanisms potentially explaining findings in mature muscles. The muscle stem cells were obtained from skeletal muscle from 14 subjects with T2D and 14 unrelated subjects with NGT which differed significantly in fasting plasma glucose levels and glucose tolerance (Additional file 1: Supplemental Table 1, [14]).

### Differentially expressed sets of related genes in skeletal muscle from subjects with T2D

#### Gene sets in muscle from MZ twins discordant for T2D

**Fig. 2:**
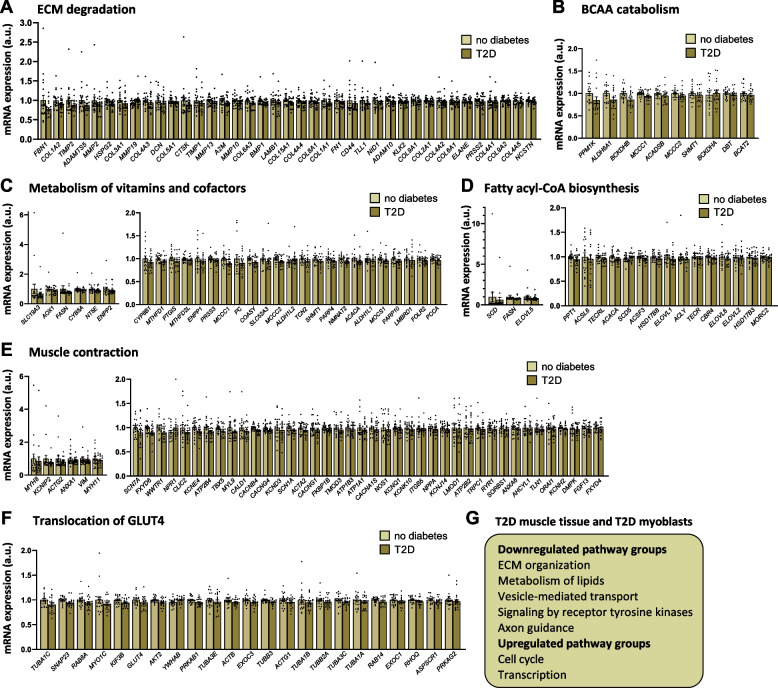
Sets of related genes with altered expression in skeletal muscle from subjects with T2D compared with subjects without T2D. Genes contributing to the significant enrichment scores of gene set enrichment analysis (GSEA, *q* < 0.10) for gene sets involved in extracellular matrix (ECM) degradation (**A**), branched-chain amino acid (BCAA) catabolism (**B**), metabolism of vitamins and cofactors (**C**), fatty acyl-CoA biosynthesis (**D**), muscle contraction (**E**), and translocation of SLC2A4 (GLUT4) to the plasma membrane (**F**), in skeletal muscle of twins with T2D versus co-twins without T2D. **G** Pathway groups found to be downregulated in the GSEA in both skeletal muscle of MZ twins with T2D and in myoblasts of subjects with T2D compared with controls. Bars in **A**–**F** represent the mean ± SEM with the mean expression levels for all mRNAs in subjects without T2D set to 1. a.u., arbitrary units. For Affymetrix expression data, while log2 intensity values after RMA data processing and normalization were used for all statistical analyses, unlogged individual expression values are plotted in the figures. Gene set enrichment analysis (GSEA) used the respective gene’s *t*-statistics for the full transcriptome data set

We first tested whether sets of related genes had altered mRNA expression in MZ twins with T2D versus co-twins without T2D. Here, GSEA yielded 22 gene sets with downregulated expression and 4 gene sets with upregulated expression in skeletal muscle from twins with T2D versus co-twins without T2D (*q* < 0.10 based on false discovery rate (FDR) analysis [25]; Table 2 and Fig. 1B). Downregulated gene sets include pathways involved in extracellular matrix (ECM) organization, branched-chain amino acid (BCAA) catabolism, metabolism of vitamins and cofactors, lipid metabolism, muscle contraction, signaling by receptor tyrosine kinases, axon guidance, and translocation of SLC2A4 (GLUT4) to the plasma membrane; and upregulated gene sets include pathways involved in the cell cycle and lipid metabolism. Individual genes contributing to the enrichment for significant gene sets are presented in Additional file 1: Supplemental Table 2. Moreover, individual genes in selected downregulated gene sets representing dysregulated pathways are shown in Fig. 2A–F.

**Table 2:** Gene sets with differential expression in skeletal muscle of twins with T2D compared with co-twins without T2D (gene set enrichment analysis with *q* < 0.1)

Pathway	Pathway group	Regulation	NES	*P*	*q*	Dysregulated miRNAs with enriched number of targets in pathway
Non-integrin membrane-ECM interactions	Extracellular matrix organization	Down	− 2.23	0.0020	0.091	
Collagen biosynthesis and modifying enzymes	Extracellular matrix organization	Down	− 2.06	0.0020	0.091	
Branched-chain amino acid catabolism	Metabolism of amino acids and derivatives	Down	− 2.05	0.0019	0.091	
Crosslinking of collagen fibrils	Extracellular matrix organization	Down	− 2.01	0.0019	0.091	
MET activates PTK2 signaling	Signal transduction	Down	− 1.98	0.0019	0.091	
ECM proteoglycans	Extracellular matrix organization	Down	− 1.98	0.0020	0.091	
Assembly of collagen fibrils and other multimeric structures	Extracellular matrix organization	Down	− 1.95	0.0020	0.091	
Collagen formation	Extracellular matrix organization	Down	− 1.95	0.0020	0.091	
Extracellular matrix organization	Extracellular matrix organization	Down	− 1.89	0.0021	0.091	
Collagen chain trimerization	Extracellular matrix organization	Down	− 1.87	0.0020	0.091	
Syndecan interactions	Extracellular matrix organization	Down	− 1.86	0.0019	0.091	miR-4797-5p
Laminin interactions	Extracellular matrix organization	Down	− 1.83	0.0019	0.091	
Fatty acyl-CoA biosynthesis	Metabolism of lipids	Down	− 1.82	0.0020	0.091	
Translocation of SLC2A4 (GLUT4) to the plasma membrane	Vesicle-mediated transport	Down	− 1.72	0.0020	0.091	miR-4643, miR-4804-3p, miR-548z
Degradation of the extracellular matrix	Extracellular matrix organization	Down	− 1.67	0.0021	0.091	
Metabolism of vitamins and cofactors	Metabolism	Down	− 1.64	0.0020	0.091	
Metabolism of water-soluble vitamins and cofactors	Metabolism	Down	− 1.64	0.0020	0.091	
Integrin cell surface interactions	Extracellular matrix organization	Down	− 1.63	0.0020	0.091	
Diseases of glycosylation		Down	− 1.57	0.0021	0.091	
Muscle contraction	Muscle contraction	Down	− 1.55	0.0020	0.091	miR-4658, miR-3936
Signaling by receptor tyrosine kinases	Signal transduction	Down	− 1.4	0.0021	0.091	miR-607, miR-623, miR-1244, miR-4494, miR-338-3p, miR-507, miR-4675, miR-4643, miR-582-5p, miR-4694-3p, miR-3148, miR-4804-5p, miR-4691-5p, miR-548z, miR-3936, miR-4717-5p, miR-2355-5p, miR-4477b, miR-4797-5p, miR-4725-5p, miR-548v
Axon guidance	Developmental biology	Down	− 1.34	0.0021	0.091	miR-623, miR-1244, miR-507 miR-485-3p, miR-4483, miR-4675, miR-4643, miR-4677-3p, miR-582-5p, miR-802, miR-3148, miR-3688-3p, miR-4658, miR-4691-5p, miR-3936, miR-4717-5p, miR-3136-3p, miR-2355-5p, miR-4797-5p, miR-548v, miR-520c-5p, miR-620, miR-1200
PPARA activates gene expression	Metabolism of lipids	Up	1.86	0.0020	0.091	miR-485-3p, miR-3148, miR-548z
Regulation of lipid metabolism by peroxisome proliferator-activated receptor alpha (PPARalpha)	Metabolism of lipids	Up	1.83	0.0020	0.091	miR-485-3p, miR-3148, miR-548z
RUNX1 regulates genes involved in megakaryocyte differentiation and platelet function	Gene expression (transcription)/RNA polymerase II transcription	Up	1.71	0.0020	0.091	miR-4483
Meiosis	Cell cycle	Up	1.6	0.0020	0.091	miR-4483

In this table, miRNAs are depicted that are differentially expressed in twins with T2D compared with co-twins without T2D (*P* < 0.05), and that have significantly more predicted targets in the pathway than expected by chance (*q* < 0.05 based on false discovery rate (FDR) analysis, identified in the miRPath database [20])

*NES* normalized enrichment score

#### Comparison with gene sets in myoblasts and myotubes of individuals with T2D or NGT

Then, we tested whether sets of related genes were also altered in myoblasts and myotubes from subjects with T2D versus NGT controls and compared these results with the gene sets identified in muscle from the MZ twin pairs discordant for T2D (Table 2 and Additional file 1: Supplemental Table 1). The GSEA yielded 81 significant gene sets with downregulated expression and 225 gene sets with upregulated expression in myoblasts from subjects with T2D versus controls (*q* < 0.05; Additional file 1: Supplemental Table 3). Of note, downregulated gene sets in myoblasts from subjects with T2D include pathways involved in, i.e., ECM organization, metabolism of lipids, vesicle-mediated transport, signaling by receptor tyrosine kinases, and axon guidance, which are pathway groups also downregulated in skeletal muscle from MZ twins with T2D (Fig. 2G). Upregulated gene sets in myoblasts from subjects with T2D are involved in, i.e., the cell cycle and transcription, which are pathway groups also upregulated in skeletal muscle from MZ twins with T2D (Fig. 2G). Moreover, the GSEA yielded 11 significant gene sets with downregulated expression and 1 gene set with upregulated expression in myotubes from subjects with T2D versus controls (*q* < 0.05; Additional file 1: Supplemental Table 4). Downregulated gene sets in myotubes from subjects with T2D are involved in, i.e., metabolism of lipids and signaling by receptor tyrosine kinases, which are pathway groups also downregulated in skeletal muscle from the MZ twins with T2D.

Importantly, we show that alterations in expression of genes regulating metabolism and ECM organization are found both in skeletal muscle from MZ twins with T2D and in cultured myoblasts from subjects with T2D (Fig. 2G).

### Differentially expressed individual genes in skeletal muscle from subjects with T2D

#### Expression in muscle from MZ twins discordant for T2D

**Fig. 3:**
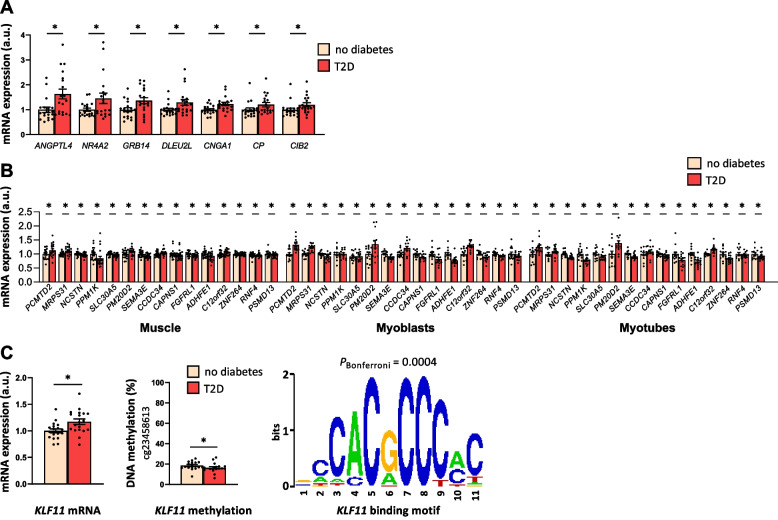
Individual genes with altered expression in skeletal muscle from subjects with T2D compared with subjects without T2D. **A** Individual genes with the largest expression differences between twins with T2D and co-twins without T2D (*P* < 0.05, same direction in more than 78% (≥ 15/19) of the discordant twin pairs and absolute difference ≥ 20%). **B** The fifteen genes which overlap and exhibit differential expressions in the same direction in muscle tissue from MZ twins with T2D, as well as in myoblasts and myotubes from subjects with T2D, compared with the respective controls. **C** KLF11 was identified as a transcription factor with increased gene expression and decreased DNA methylation as well as significant enrichment of binding motifs in promoter regions when analyzing all differentially expressed genes in twins with T2D versus co-twins without T2D. To the right are the Bonferroni adjusted *P* value and the binding motif for KLF11. The bars represent the mean ± SEM with the mean expression levels for all mRNAs set to 1 in subjects without T2D. For mRNA expression data, while log2 intensity values after RMA data processing and normalization were used for all statistical analyses, unlogged individual expression values are plotted in the figures. *P < 0.05, a.u., arbitrary units

We next tested whether the expression of individual genes was altered in skeletal muscle from MZ twins with T2D versus co-twins without T2D. We found that 863 (4%) of 21,732 analyzed probe sets annotated to transcripts, and corresponding to 823 unique genes, had altered expression in muscle from twins with T2D based on nominal *P* values < 0.05 (Additional file 1: Supplemental Table 5 and Additional file 1: Supplemental Fig. 1A). None of these differences remained significant after FDR correction using the Benjamini–Hochberg method. Although no transcripts had *q* < 0.05 based on FDR analysis, the expression difference for 277 of the 863 transcripts with *P* < 0.05 went in the same direction for more than 78% (≥ 15/19) of the twin pairs discordant for T2D (marked in Additional file 1: Supplemental Table 5). The expression differences of these transcripts range from − 20 to 63% in muscle from twins with T2D versus co-twins without T2D. Genes with the largest expression differences between twins with T2D and co-twins without T2D (*P* < 0.05, same direction in ≥ 15/19 twin pairs and absolute difference ≥ 20%) are presented in Fig. 3A and include genes with known functions in muscle and/or glucose/lipid metabolism such as *ANGPTL4*, *NR4A2* (*NURR1*), and *GRB14* [26–28].

#### Comparison with expression in muscle of unrelated individuals with T2D, IGT, or NGT

We next analyzed expression of the genes found to be differentially expressed in muscle from the T2D discordant twin pairs based on nominal *P* values (*P* < 0.05, Additional file 1: Supplemental Table 5) in skeletal muscle from 15 subjects with T2D or IGT and 362 NGT controls using gene expression array data [12]. We found that 17 genes with differential expression in skeletal muscle of the discordant twins were also altered in muscle from the 15 subjects with T2D/IGT versus the 362 NGT controls (*q* < 0.05, Additional file 1: Supplemental Table 5 and Additional file 1: Supplemental Fig. 1B). The overlapping genes include *MTHFD1* and *SETBP1*, encoding proteins involved in DNA methylation and histone methylation regulation, respectively, as well as *DBNDD1*, *SLC47A2*, *STK35*, and *TMEM222* (Additional file 1: Supplemental Fig. 1C).

#### Comparison with expression in myoblasts and myotubes of individuals with T2D or NGT

Furthermore, we analyzed expression of the genes found to be differentially expressed in muscle from the T2D discordant twin pairs based on nominal *P* values (*P* < 0.05, Additional file 1: Supplemental Table 5) in myoblasts and myotubes obtained from 13 T2D and 13 NGT unrelated subjects using gene expression array data [14]. These analyses were performed to test if the abnormalities found in muscle from the MZ twins with T2D (i) may exist already at stem cell and progenitor stage and (ii) may imply a metabolic memory, since the myoblasts and myotubes from individuals with and without diabetes are cultured in the same conditions. Here, we found 73 genes to exhibit differential expression in both skeletal muscle from twins with T2D and in myoblasts from subjects with T2D based on nominal *P* < 0.05 (Additional file 1: Supplemental Table 6 and Additional file 1: Supplemental Fig. 1B), including genes encoding proteins involved in regulation of insulin sensitivity (i.e., *EGR2* and *FABP3*) and muscle growth (*FSD1* and *IGFBP3*). We further found 29 of the analyzed genes to exhibit differential expression in both skeletal muscle from twins with T2D and in myotubes from subjects with T2D when compared with the respective controls based on nominal *P* < 0.05 (Additional file 1: Supplemental Table 7 and Additional file 1: Supplemental Fig. 1B), including *MTHFD1* and *TXNIP*, the latter was previously shown to be upregulated in skeletal muscle from subjects with prediabetes and T2D and it was inversely correlated with glucose uptake [29]. Fifteen genes overlapped and exhibited differential expression in skeletal muscle from MZ twins, myoblasts and myotubes in the same direction in subjects with T2D compared with the respective controls (Fig. 3B and Additional file 1: Supplemental Fig. 1B). These genes include *PCMTD2* (involved in histone methylation), *PPM1K* (involved in BCAA catabolism), and *MRPS31* (involved in mitochondrial metabolism) (Additional file 1: Supplemental Fig. 1C).

Overall, 99 unique genes with differential expression in muscle from T2D discordant twins could be validated in either skeletal muscle or cultured myoblasts or myotubes from unrelated subjects with T2D versus controls (Additional file 1: Supplemental Table 5).

### Enrichment of transcription factor binding sites in promoter regions of differentially expressed genes

The binding of transcription factors to promoter regions is an important mechanism by which expression is regulated. To get hints on which factors could be responsible for the patterns of expression observed, we searched for over-representation of DNA binding motifs in promoter regions of the differentially expressed genes in skeletal muscle from MZ twins discordant for T2D presented in Additional file 1: Supplemental Table 5 using Pscan [30] and the JASPAR database [31]. We found a significant enrichment of recognition sequences for 44 transcription factors including several members of the EGR, KLF, and SP transcription factor families (*P*_*Bonferroni*_ < 0.05, Additional file 1: Supplemental Table 8). Among these, the genes *EGR2* and *KLF11* also showed increased mRNA expression in the skeletal muscle of MZ twins with T2D (*P* < 0.05, *KLF11* is presented in Fig. 3C).

### Differential DNA methylation in skeletal muscle from subjects with T2D

#### Methylation in muscle from MZ twins discordant for T2D

**Fig. 4:**
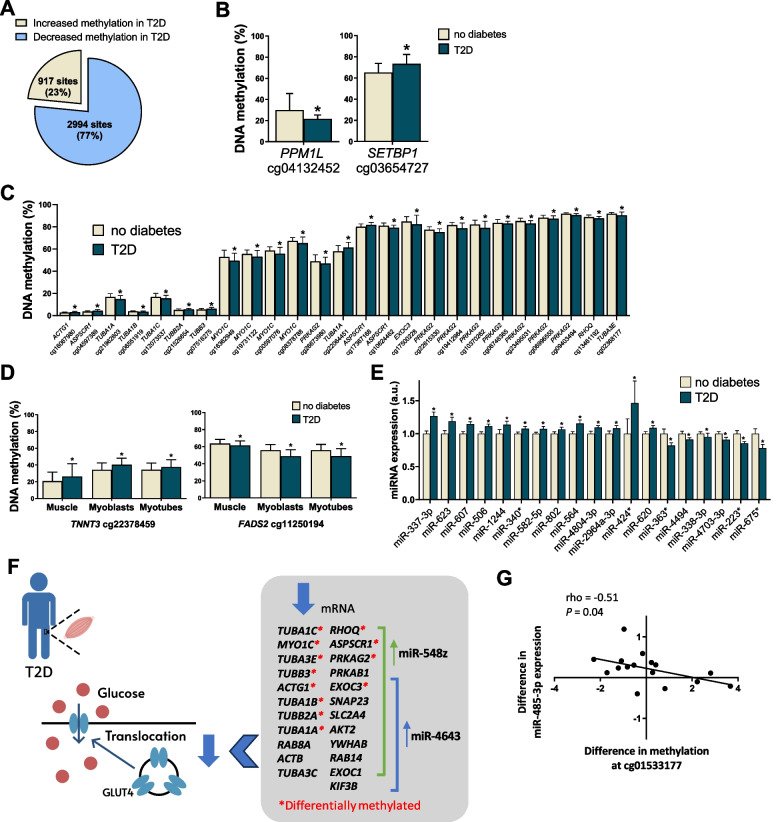
Differential DNA methylation and miRNA expression in skeletal muscle from subjects with T2D compared with subjects without T2D. **A** Pie chart describing the number (and percentage) of sites that exhibit increased or decreased DNA methylation in skeletal muscle from MZ twins with T2D compared with co-twins without T2D (sites with *P* < 0.05 and methylation differences in the same direction for more than 82% (≥ 14/17) of the discordant twin pairs). **B** Differential DNA methylation of cg04132452 in the body of *PPM1L* and cg03654727 in the 3′UTR of *SETBP1* in muscle of MZ twins with T2D versus co-twins without T2D. **C** Differentially methylated genes in the “translocation of SLC2A4 (GLUT4) to the plasma membrane” pathway in skeletal muscle of MZ twins with T2D versus co-twins without T2D. **D** CpG sites in *TNNT3* and *FADS2* showing differential DNA methylation in the same direction in muscle tissue from MZ twins with T2D, as well as in myoblasts and myotubes from subjects with T2D, compared with the respective controls. **E** miRNAs with expression differences between MZ twins with T2D and co-twins without T2D (*P* < 0.05 and the expression difference went in the same direction for more than 78% (≥ 15/19) of the discordant twin pairs). **F** A cartoon summarizing some of the findings in this multiomics study, linking altered miRNA, DNA methylation, and mRNA expression levels in skeletal muscle of MZ twins discordant for T2D to the GLUT4 translocation pathway. miR-4643 and miR-548z are upregulated in T2D twins (*P* < 0.05) and have more predicted targets than expected in the GLUT4 translocation pathway (*q* < 0.05). The 23 presented genes in the box are the genes contributing to a significantly downregulated “translocation of SLC2A4 (GLUT4) to the plasma membrane” pathway, identified by GSEA (*q* < 0.10). At the same time, 13 of the 23 genes have one or more CpG site(s) with differential DNA methylation between twins with T2D versus co-twins without T2D (*P* < 0.05). **G** The twin pair differences in log2 expression levels of miR-485-3p correlated negatively with twin pair differences in methylation levels of cg01533177 upstream of the transcription start site of the miR-485 gene. Within–twin-pair differences of the measures were calculated by subtracting the value of the co-twin without T2D from the value of the T2D co-twin (*n* = 16 twin pairs with both miRNA and DNA methylation array data). DNA methylation data in **B**–**D** are shown as mean ± SD. The bars in **E** represent the mean ± SEM with the mean expression levels for all miRNAs set to 1 in subjects without T2D. For miRNA expression data, while log2 intensity values after RMA data processing and normalization were used for all statistical analyses, unlogged individual expression values are plotted in the figures. *P < 0.05, a.u., arbitrary units

To investigate epigenetic dysregulation in skeletal muscle, we further analyzed DNA methylation in the discordant twins using Infinium arrays. We found that 15,647 individual CpG sites had differential methylation in skeletal muscle from twins with T2D compared with co-twins without T2D at nominal *P* < 0.05 (Additional file 1: Supplemental Table 9A). None of these differences remained significant after FDR correction using the Benjamini–Hochberg method. Nevertheless, for 3911 of the sites with *P* < 0.05, the methylation differences went in the same direction for more than 82% (≥ 14/17) of the twin pairs discordant for T2D (marked in Additional file 1: Supplemental Table 9A). The majority of these CpG sites (2994 sites; 77%) displayed decreased DNA methylation in twins with T2D (Fig. 4A). We further tested if T2D was associated with alterations in the average DNA methylation levels in different gene and CpG island regions using Illumina’s annotations [18] and found no differences in muscle from twins with T2D compared with non-diabetic co-twins (Additional file 1: Supplemental Table 9B).

The biggest absolute differences in DNA methylation levels of individual CpG sites were found in cg04132452 in the body of *PPM1L* and in cg03654727 in the 3′UTR of *SETBP1* (*P* < 0.05, Fig. 4B). At the same time, the mRNA expression of *PPM1L* was increased and expression of *SETBP1* was decreased in MZ twins with T2D compared with co-twins without T2D (*P* < 0.05, Additional file 1: Supplemental Table 5).

DNA methylation may account for altered mRNA levels, and for that reason we tested if genes contributing to the significant GSEA results presented in Table 2 and Additional file 1: Supplemental Table 2 also were differentially methylated in skeletal muscle from MZ twins with T2D in comparison to co-twins without T2D. Indeed, 245 (48.4%) of the 506 genes presented in Additional file 1: Supplemental Table 2 had altered DNA methylation patterns in muscle from twins with T2D versus co-twins without T2D based on nominal *P* values (*P* < 0.05, Additional file 1: Supplemental Table 9A). Differentially methylated genes in the translocation of GLUT4 to the plasma membrane pathway in skeletal muscle of MZ twins with T2D versus co-twins without T2D are presented in Fig. 4C.

Next, we tested whether the genes with the largest differences in expression (*P* < 0.05, same direction in ≥ 15/19 twin pairs and absolute difference ≥ 20%; Fig. 3A) were also differentially methylated in muscle from twins with T2D compared with co-twins without T2D. Two of the top genes (*ANGPTL4* and *NR4A2*) were annotated to one and two CpG sites with differential methylation, respectively (*P* < 0.05, Additional file 1: Supplemental Table 9A). Among the two differentially expressed genes encoding transcription factors with enrichment of binding sites in the promoters of dysregulated genes, cg23458613 in the TSS1500 of *KLF11* displayed reduced DNA methylation in twins with T2D compared with co-twins without T2D (*P* < 0.05, Fig. 3C).

#### Comparison with methylation in myoblasts and myotubes from individuals with T2D or NGT

Next, we analyzed DNA methylation of the 15,647 CpG sites showing differential DNA methylation in muscle from twins discordant for T2D based on nominal *P* values (*P* < 0.05, Additional file 1: Supplemental Table 9A) also in myoblasts and myotubes obtained from 14 NGT and 14 T2D unrelated subjects. Here, we found 608 sites to exhibit differential methylation in the same direction in both muscle tissue and myoblasts and 406 sites to exhibit differential methylation in the same direction in both muscle tissue and myotubes from subjects with T2D versus controls (nominal *P* < 0.05, Additional file 1: Supplemental Tables 10 and 11). Moreover, 130 sites annotated to 99 unique genes overlapped and exhibited differential methylation in muscle tissue, myoblasts, and myotubes in the same direction when comparing subjects with T2D versus controls (marked in Additional file 1: Supplemental Table 9A). These sites include, i.e., cg22378459 in *TNNT3*, the gene encoding the fast skeletal muscle troponin T, and cg11250194 in *FADS2*, encoding fatty acid desaturase 2 (Fig. 4D).

### Differential miRNA expression in skeletal muscle from subjects with T2D

#### miRNA levels in muscle from MZ twins discordant for T2D

We proceeded to analyze expression of 1733 miRNAs in skeletal muscle obtained from 19 of the MZ twin pairs discordant for T2D. Sixty-nine miRNAs (4% of those analyzed) were differentially expressed in the twin pairs at nominal *P* < 0.05 (Additional file 1: Supplemental Table 12). None of these differences remained significant after FDR correction using the Benjamini–Hochberg method. Nevertheless, for 19 of the 69 miRNAs with *P* < 0.05, the expression difference went in the same direction for more than 78% (≥ 15/19) of the MZ twin pairs discordant for T2D (Fig. 4E, marked in Additional file 1: Supplemental Table 12). Six of these miRNAs were downregulated and 13 were upregulated in the twins with T2D. Additionally, 15 of these 69 miRNAs have previously been linked to muscle function, based on a systematic PubMed search using the search terms “skeletal muscle” and each respective miRNA (Additional file 1: Supplemental Table 12).

Since miRNAs within the same family often target the same mRNAs, we examined whether any of the nominally differentially expressed miRNAs in discordant twins represented the same family/families as defined by miRBase (release 22.1) [32]. Indeed, five miRNA families, the miR-322, miR-506, miR-515, miR-548, and miR-551 families, were represented by two differentially expressed miRNAs each (Additional file 1: Supplemental Table 12).

Further, intra–twin-pair correlations revealed that the differences in expression levels of eight of the 69 nominally dysregulated miRNAs (miR-154*, miR-1244, miR-3184, miR-548v, miR-497, miR-130a*, miR-135b*, and miR-let7g*) also correlated with differences between co-twins in fasting glucose levels, 2-h glucose measures in an oral glucose tolerance test (OGTT) and/or HbA1c levels (*P* < 0.05, Additional file 1: Supplemental Table 13).

#### Predicted targets of the differentially expressed miRNAs in muscle from MZ twins discordant for T2D

To find out if any of the differentially expressed miRNAs are enriched (have significantly more predicted targets than expected by chance) for the genes included in the pathways identified to be dysregulated in the GSEA of mRNA expression in muscle presented in Table 2 and Additional file 1: Supplemental Table 2, we used the miRPath database [20], which includes both experimentally validated and predicted targets. Nine of the 26 significant pathways have one or more differentially expressed miRNA(s) with significantly more predicted targets in the pathway than expected by chance (*q* < 0.05 based on FDR analysis, Table 2). These include miR-4643 and miR-548z, predicted to regulate genes involved in GLUT4 translocation (Fig. 4F), as well as miR-4658 and miR-3936 predicted to regulate genes involved in muscle contraction, and miR-607 predicted to regulate genes involved in signaling by receptor tyrosine kinases (Table 2).

We then used the multiMiR tool (http://multimir.ucdenver.edu/) which integrated miRTarBase, TarBase, DIANA-microT, MicroCosm, miRanda, miRDB, PITA, ELMO, TargetScan, PicTar, and miRecords, to find predicted targets for the 69 differentially expressed miRNAs presented in Additional file 1: Supplemental Table 12, and we overlapped the predicted targets with the 823 differentially expressed genes presented in Additional file 1: Supplemental Table 5. Here, we found that 569 (69.1%) of the 823 differentially expressed genes are predicted targets for the 69 differentially expressed miRNAs (Additional file 1: Supplemental Table 14).

#### DNA methylation of the differentially expressed miRNAs in muscle from MZ twins discordant for T2D

Since miRNA expression also could be regulated by epigenetic modifications, we analyzed DNA methylation of CpG sites annotated to the analyzed miRNA genes (based on Illumina manifest and genome build 37) in skeletal muscle from the twin pairs discordant for T2D. A total of 175 methylation sites, which are included on and could be analyzed with the Illumina Infinium HumanMethylation450 Bead-Chip, were annotated to the 69 miRNAs with nominally differential expression in discordant twins (*P* < 0.05). Four of these sites, annotated to miR-135b, miR-223, and miR-675, exhibited decreased methylation in the twins with T2D compared with co-twins without T2D (nominal *P* < 0.05, Additional file 1: Supplemental Table 9A). One of these sites, cg09701145 in miR-675, also displayed decreased methylation in T2D myotubes compared with NGT myotubes (Additional file 1: Supplemental Table 11). Further, intra–twin-pair correlations revealed that the differences in expression levels of seven of the 69 identified dysregulated miRNAs correlated with differences in methylation levels of one or more CpG site(s) in/near the gene (*P* < 0.05, Additional file 1: Supplemental Table 15, example in Fig. 4G). Interestingly, three of the seven miRNAs correlating with DNA methylation (miR-485-3p, miR-620, and miR-338-3p) are enriched (have significantly more predicted targets than expected by chance) for genes included in dysregulated pathways identified in the GSEA of mRNA expression in muscle of the MZ twins presented in Table 2. These include pathways involved in axon guidance, signaling by receptor tyrosine kinases, and lipid metabolism.

Together, these data suggest that combinations of DNA methylation and miRNAs contribute to dysregulation of gene expression in skeletal muscle from individuals with T2D.

### Functional validation of selected candidate miRNAs in cultured human myotubes

**Fig. 5:**
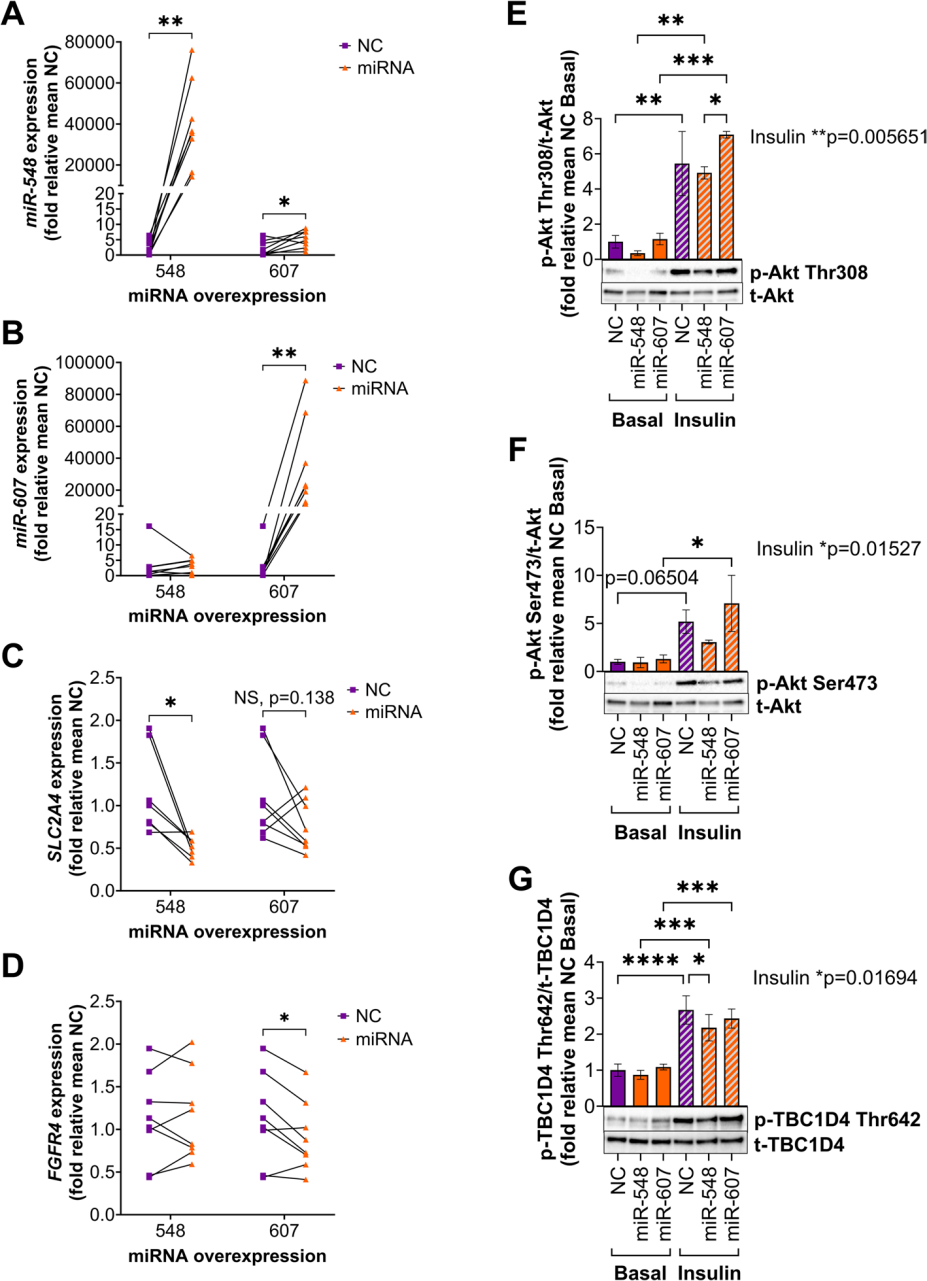
miR-548 and miR-607 overexpression in primary human muscle cells altered expression of target genes and miR-548 impaired insulin signaling. **A**–**G** miR-548 and miR-607 were overexpressed using miRNA mimics in human muscle cells differentiated in vitro. Samples were collected after stimulation with 100 nM insulin for 30 min or in the basal state from *n* = 4 different passages. **A**–**D** miRNA and mRNA expression were determined by qPCR. Insulin stimulation had no overall effect on RNA expression compared to the basal state, as determined by two-way repeated measures ANOVA (insulin vs. basal, *P* = 0.0826 [*miR-548*], *P* = 0.9399 [*miR-607*], *P* = 0.2842 [*SLC2A4*], *P* = 0.9890 [*FGFR4*]). Consequently, the basal and insulin-stimulated samples for each group were merged for RNA expression analysis. Expression of *miR-548* (**A**), *miR-607* (**B**), *SLC2A4* (encoding GLUT4) (**C**), and *FGFR4* (**D**) relative to mean negative control (NC). The expression of *miR-548* (mean ± SEM) was 2.2 ± 0.8, 39,562 ± 7460, and 5.2 ± 1.0 in the NC, miR-548, and miR-607 groups, respectively (*n* = 8 experimental replicates). The expression of *miR-607* (mean ± SEM) was 3.1 ± 1.9, 3.1 ± 0.8, and 35,313 ± 10,034 in the NC, miR-548, and miR-607 groups, respectively (*n* = 8 experimental replicates). The expression of *SLC2A4* (mean ± SEM) was 1.15 ± 0.19 and 0.51 ± 0.05 in the NC vs. miR-548 groups (*n* = 7 experimental replicates), and 1.09 ± 0.18 and 0.76 ± 0.11 in the NC vs. miR-607 groups (*n* = 8 experimental replicates). The expression of *FGFR4* (mean ± SEM) was 1.12 ± 0.19, 1.16 ± 0.18, and 0.91 ± 0.15 in the NC, miR-548, and miR-607 groups, respectively. Statistical significance was determined by paired *t*-tests (NC vs. miRNA). **E**–**G** Phosphorylation of Akt (PKB) and TBC1D4 (AS160) was determined by western blotting. Phosphorylation was normalized to the protein levels of each respective protein and expressed as fold relative to mean NC basal. Phosphorylation of Akt at Thr308 (**E**) and at Ser473 (**F**), and TBC1D4 at Thr642 (**G**) (*n* = 4 independent experiments). Representative blots are shown. Statistical significance was determined by two-way repeated measures ANOVA followed by tests between the treatments (basal vs. insulin) within each group and between groups (NC vs. miR-548, NC vs. miR-607, and miR-548 vs. miR-607) within each treatment (Fisher’s LSD test)

To functionally explore the mechanisms by which miRNAs contribute to T2D pathogenesis, two miRNAs (miR-548 and miR-607) were selected for follow-up experiments in primary human muscle cells based on (i) being upregulated in muscle from twins with T2D versus co-twins without T2D (Fig. 4E and Additional file 1: Supplemental Table 12) and (ii) having significantly more predicted targets than expected by chance for genes found to be dysregulated in the “GLUT4 translocation” and “signaling by receptor tyrosine kinases” pathways, respectively (Table 2 and Additional file 1: Supplemental Table 2). To mirror the increased expression in muscle from MZ twins discordant for T2D, miR-548 and miR-607 were successfully overexpressed using miRNA mimics in human myotubes cultured in vitro (Fig. 5A–B). The expression of *miR-548* was significantly increased also after miR-607 overexpression (Fig. 5A). However, the *miR-548* expression is 4 orders of magnitude lower compared to the expression of miR-607 suggesting that the impact of increased miR-548 in this condition is likely negligible or very small. First, we analyzed the expression of selected genes that contributed to the downregulated pathways in muscle from twins with T2D (Fig. 2F). *GLUT4* (*SLC2A4*), the insulin-responsive glucose transporter, which was among the downregulated genes in the “GLUT4 translocation” pathway and a predicted target for miR-548 (Table 2), showed indeed significantly decreased expression after *miR-548* overexpression in myotubes (Fig. 5C). Additionally, *FGFR4*, which was among the downregulated genes in the “signaling by receptor tyrosine kinases” pathway and a predicted target for miR-607 (Table 2 and Additional file 1: Supplemental Table 2) displayed a significant decrease in expression after *miR-607* overexpression in myotubes (Fig. 5D). We proceeded to examine whether overexpression of *miR-548* or *miR-607* could also directly impact the activation of key proteins involved in insulin signaling and glucose uptake in human myotubes using western blotting with phospho-specific antibodies. First, we monitored the phosphorylation of Akt at the activating sites Thr308 and Ser473. As expected, insulin induced an overall increase in Akt phosphorylation of Thr308 (Fig. 5E), but there were no significant differences in the specific phosphorylation relative to the negative control (NC) after miRNA overexpression of either *miR-548* or *miR-607*. For Ser473 on Akt, insulin induced phosphorylation for the NC (*P* = 0.065) and for myotubes overexpressing *miR-607* (*P* ≤ 0.05), but insulin could not induce Ser473 phosphorylation in myotubes overexpressing *miR-548*, supporting an impaired activation of Akt when this miRNA is increased (Fig. 5F). Additionally, the fold induction in Akt phosphorylation in response to insulin was not significantly altered after miRNA overexpression indicating intact insulin sensitivity (data not shown). Next, we monitored TBC1D4, a downstream target of Akt controlling GLUT4 translocation. Interestingly, the insulin-induced phosphorylation of TBC1D4 at Thr642 was significantly lower in the miR-548-expressing cells compared to the NC (Fig. 5G). As the degree of TBC1D4 phosphorylation is previously known to correlate with the degree of glucose uptake, these data indicate that the level of insulin-induced uptake of glucose is decreased after *miR-548* overexpression. Together, this suggests that miRNAs dysregulated in muscle from individuals with T2D likely contribute to impaired glucose uptake by decreasing both *GLUT4* (*SLC2A4*) expression as well as insulin signaling, and potentially GLUT4 translocation to the plasma membrane.

## Discussion

T2D is a complex disease caused by an interplay between genetic, epigenetic, and environmental factors [33]. In this study, we performed an integrative multiomics analysis to provide new clues of the underlying regulatory mechanisms of T2D. Skeletal muscle biopsies from MZ twin pairs discordant for T2D were investigated at the gene expression, DNA methylation, and miRNA expression levels (Additional file 1: Supplemental Fig. 1C). The unique study design of disease-discordant MZ twin pairs enables to control for many potential confounders encountered in general population studies such as differences in genetic background, early-life environmental exposure, age, and sex. The identified differences in mRNA expression of individual genes between MZ twins with T2D compared with co-twins without T2D were modest. However, modest changes in expression of individual genes may have an important biological impact when combined for an entire pathway. Indeed, the GSEA identified several dysregulated pathways with relevance to T2D and muscle function. Interestingly, twelve of the 22 significantly downregulated pathways in muscle from twins with T2D are involved in ECM organization. Enrichment of downregulated genes involved in ECM organization was also seen in myoblasts from subjects with T2D compared with unrelated NGT subjects. Skeletal muscle ECM remodeling is thought to be a feature of the pathogenic milieu associated with metabolic dysregulation, obesity, and eventual diabetes [34]. It can lead to an increased physical barrier for insulin and glucose transport, decreased vascular insulin delivery, and decreased integrin signaling leading to decreased insulin signaling [35]. Of note, miR-4797-5p had differential expression in muscle from twins with T2D (nominal *P* < 0.05) and has significantly more potential targets in ECM remodeling than expected by chance. We also found downregulation of genes involved in BCAA catabolism in skeletal muscle from twins with T2D. This agrees with a study by Sjögren et al. [36]. Circulating levels of BCAAs are known to be elevated in subjects with T2D [37]. Skeletal muscle is the major contributor to systemic BCAA oxidation [38] and pathologic levels of BCAAs can disturb both insulin sensitivity and secretion [39]. We found that the gene encoding PPM1K was downregulated in muscle tissue, myoblasts, and myotubes from subjects with T2D compared to the respective control. PPM1K is involved in reducing circulating BCAAs and it increases insulin sensitivity [40].

In the present study, we investigated, for the first time, both the transcriptome and methylome in skeletal muscle from MZ twin pairs discordant for T2D. We wanted to find out if genes/pathways with differential expression in twins with T2D also exhibit differential DNA methylation patterns. We and others have previously proposed strong genetic effects on the DNA methylation pattern [41–44], and this could be an explanation to the subtle differences found both in this study and a previous study [11] when comparing MZ twin pairs. However, some of the findings in discordant twins were also seen in myoblasts and/or myotubes from unrelated subjects with T2D and NGT, supporting the idea that DNA methylation affects the phenotype in skeletal muscle from discordant twins too. A CpG site in *SETBP1*, which encodes the SET-binding protein 1, had the largest increase in DNA methylation in muscle from twins with T2D compared with non-T2D co-twins, and this gene also showed decreased mRNA expression in skeletal muscle from subjects with T2D, both in the twin cohort and the validation cohort including unrelated subjects [12]. Differential DNA methylation of a site linked to *SETBP1* has previously been associated with obesity [45]. SET-binding protein 1 has been shown to form a multiprotein complex resulting in increased chromatin accessibility and transcriptional activation [46]. Moreover, a CpG site in *PPM1L*, encoding protein phosphatase Mg^2+^/Mn^2+^ dependent 1L, had the largest decrease in DNA methylation in muscle from twins with T2D compared with non-T2D co-twins, and this gene also showed increased expression in muscle from twins with T2D. *PPM1L* has previously been linked to traits associated with the metabolic syndrome [47].

In addition, about half of the genes contributing to the enrichment of significant gene sets in the GSEA had differential DNA methylation in twins with T2D in comparison to co-twins without T2D (nominal *P* < 0.05). This suggests that DNA methylation could be one mechanism behind the dysregulation although it must be validated in larger studies. The vast majority of the nominally differentially methylated CpG sites showed decreased DNA methylation in skeletal muscle from twins with T2D compared with the co-twins without T2D. Intriguingly, we have previously observed similar epigenetic patterns in both liver and pancreatic islets in which 94 and 97%, respectively, of differentially methylated CpG sites showed decreased DNA methylation in subjects with T2D compared with controls [48, 49]. The hypomethylation of individual CpG sites may be explained by changed expression and/or activity of proteins controlling DNA methylation, or a methyl group deficiency [50]. We have previously observed significantly reduced circulating folate levels in subjects with T2D compared with controls [49], which may partly explain why most significant sites in subjects with T2D are hypomethylated. In this study, we added new pieces of the puzzle by observing downregulation of genes involved in metabolism of vitamins and cofactors, including the folate receptor encoding gene *FOLR2*, in muscle from twins with T2D. Also, *MTHFD1*, encoding methylenetetrahydrofolate dehydrogenase 1, was downregulated in muscle from twins with T2D, in subjects with T2D and IGT in the validation study, and in cultured myotubes from subjects with T2D. Altered activity of MTHFD1 may affect the ability to deal with folate deficiency [51] and polymorphisms in *MTHFD1* have been shown to lead to genome-wide decrease in DNA methylation [52]. Some genes included in the enriched pathways with downregulated expression, e.g., translocation of GLUT4 to the plasma membrane, also showed reduced methylation of CpG sites in muscle from twins with T2D. Traditionally, increased DNA methylation was thought to reduce gene expression [53], and indeed this is often the case when methylation takes place in promoter regions where methylation can sterically hinder transcription factors from binding or attract methyl binding proteins which subsequently can attract, e.g., HDACs and co-repressors, closing down the chromatin structure. However, we and others have also shown that increased DNA methylation can be linked to increased gene expression, for example, when methylation takes place in the gene body or in CpG sites where transcriptional repressors binds, as well as in promoters of miRNA, thereby reducing miRNA expression and subsequently increasing expression of gene targets [9, 33, 54]. Indeed, 16 of the 19 CpG sites with lower methylation in muscle from twins with T2D and being annotated to genes which are part of the downregulated GLUT4 translocation pathway are in the gene body, while 5 of 6 CpG sites with lower methylation in muscle from twins with T2D and being annotated to genes which are part of the upregulated “regulation of lipid metabolism by peroxisome proliferator-activated receptor alpha (PPARalpha)” pathway are in promoter regions.

It is a complex task linking dysregulation of miRNAs, which have multiple targets and can cause both up- and downregulation of mRNA and/or proteins, to mechanisms of disease development. We identified 32 miRNAs with differential expression in T2D muscle (nominal *P* < 0.05) that had significantly more predicted targets than expected in pathways that were dysregulated at the mRNA level. These include miR-4643 and miR-548z, predicted to regulate genes involved in GLUT4 translocation (Fig. 4F), miR-4658 and miR-3936 predicted to regulate genes involved in muscle contraction as well as miR-607 predicted to regulate genes involved in signaling by receptor tyrosine kinases pathways. Glucose transport is rate limiting for postprandial glucose utilization by muscle tissues [55], and both insulin signaling and muscle contraction can cause GLUT4 translocation to the plasma membrane [55]. The finding of reduced expression of genes involved in GLUT4 translocation in T2D muscle is consistent with previous results linking insulin resistance with impaired GLUT4 translocation in muscle [56]. In addition, a recent large-scale genome-wide association study identified candidate genes implicated in the expression or trafficking of GLUT4 [57]. We have previously identified miR-15b and miR-16 as downregulated in T2D muscle in a smaller subset of discordant MZ twin pairs [10]. In the present study, both miR-15b and miR-16 followed the same pattern with reduced levels in muscle of T2D subjects compared with that of control subjects although it was not statistically significant (data not shown). This could be due to, for example, phenotypic differences of the additional twin pairs, a difference in cell composition of the biopsies, or the fact that different types of arrays were used. Importantly, to mimic T2D-associated changes in muscle from the discordant twins, we overexpressed two miRNAs, miR-548 and miR-607, in cultured human myotubes. In agreement with that miR-548z is predicted to regulate genes involved in GLUT4 translocation, we found decreased expression of *GLUT4* and nominally impaired insulin-stimulated phosphorylation of Akt (Ser473) and TBC1D4 in muscle cells overexpressing miR-548. Additionally, overexpression of miR-607 reduced expression of its predicted target *FGFR4*, involved in signaling by receptor tyrosine kinases pathways.

This study has potential limitations. While we found differential expression of gene sets/biological pathways in skeletal muscle from twins with T2D compared with co-twins without T2D when correcting for multiple testing, expression of individual genes and miRNAs as well as DNA methylation did not stand the FDR analysis. However, when we inspected the mRNA, miRNA, and DNA methylation results with *P* < 0.05, we found that expression of several genes and miRNAs as well as methylation of several sites were altered in the same direction in muscle from most twins with T2D compared with the co-twins without T2D. We therefore tested if we could validate/follow up the results from the twins in two different data sets, one from skeletal muscle biopsies and one from myoblasts and myotubes from subjects with T2D and unrelated controls [12, 14]. Indeed, several findings from muscle of MZ twins discordant for T2D could be validated in these independent cohorts. Unfortunately, we lack material from muscle to also technically validate our results from the discordant twins. However, we have previously technically validated the genome-wide methods used in the present study using both RT-qPCR and pyrosequencing, and we hence believe the results from the expression and methylation arrays are technically robust [43, 48, 58]. To our knowledge, there are no EWAS studies using methylation arrays from Illumina in skeletal muscle from individuals with T2D to use for further validation [59]. However, future studies in skeletal muscle from larger cohorts of MZ twins discordant for T2D are needed to further replicate the results from the present study.

We have previously studied mRNA and miRNA expression, as well as DNA methylation in adipose tissue from MZ twins discordant for T2D [43, 60]. Interestingly, genes affecting lipid metabolism and regulation of DNA methylation, including, i.e., the genes *ELOVL5*, *ELOVL6*, *FASN*, and *MTHFD1* were downregulated in both skeletal muscle, as seen in the present study, and adipose tissue [43] from MZ twins with T2D. These data suggest that some pathways and genes are regulated in a similar fashion in both muscle and adipose tissue from subjects with T2D.

## Conclusions

In conclusion, this study provides novel insights into the underlying molecular mechanisms of skeletal muscle dysfunction in subjects with T2D. Using multiomics, we demonstrate that T2D is associated with aberrant skeletal muscle gene expression of key metabolic pathways, and we identified changes in DNA methylation and miRNA levels that may contribute to altered expression of genes in these pathways (Additional file 1: Supplemental Fig. 1C).

## Supplementary Information

Additional file 1: Supplemental Table 1 Clinical characteristics of donors of muscle cells. Supplemental Table 2 Genes contributing to the enrichment scores of significantly regulated pathways obtained from the gene set enrichment analysis in muscle tissue from twins with type 2 diabetes compared with co-twins without type 2 diabetes. Supplemental Table 3 Gene sets with differential expression in myoblasts from subjects with type 2 diabetes versus controls (gene set enrichment analysis with *q* < 0.05). Supplemental Table 4 Gene sets with differential expression in myotubes from subjects with type 2 diabetes versus controls (gene set enrichment analysis with *q* < 0.05). Supplemental Table 5 Differentially expressed genes between twins with type 2 diabetes and co-twins without diabetes (*P* < 0.05). Supplemental Fig. 1 (A) Differentially expressed genes between monozygotic (MZ) twins with type 2 diabetes (T2D) and co-twins without T2D based on nominal *P* values (*P* < 0.05). Log2 fold changes in the volcano plot are shown when comparing expression in muscle from MZ twins with versus co-twins without T2D. The dashed line indicates P < 0.05. Gene labels are based on the top 20 most significant transcripts, however of those, only 10 labels are displayed as there were no annotations for the other 10. (B) Overlap of genes with differential expression in the skeletal muscle from MZ twins discordant for T2D (*P* < 0.05), in skeletal muscle from 15 subjects with T2D or impaired glucose tolerance (IGT) versus 362 normal glucose tolerance (NGT) controls (*q* < 0.05), as well as in cultured myoblasts and myotubes from 13 T2D versus 13 NGT unrelated subjects (*P* < 0.05). These results are also presented in Supplemental Table 5. (C) Schematic summary, partly based on Fig. 1A, highlighting some key results from this study. Bold text marks findings, we could validate in muscle biopsies or cells from unrelated subjects and/or based on functional experiments overexpressing two miRNAs in cultured human myotubes. Supplemental Table 6 Differentially expressed genes in myoblasts from subjects with type 2 diabetes compared with controls among the 863 genes differentially expressed in muscle from the type 2 diabetes discordant twin pairs (*P* < 0.05). Supplemental Table 7 Differentially expressed genes in myotubes from subjects with type 2 diabetes compared with controls among the 863 genes differentially expressed in muscle from the type 2 diabetes discordant twin pairs (*P* < 0.05). Supplemental Table 8 JASPAR motif search results in the set of differentially expressed genes between twins with type 2 diabetes and co-twins without type 2 diabetes. Supplemental Table 9 (A) Differentially methylated CpG sites in muscle from twins with type 2 diabetes compared with co-twins without type 2 diabetes (*P* < 0.05). (B) Average DNA methylation levels in different gene and CpG island regions using Illumina’s annotations [18] in muscle from twins with T2D compared with non-diabetic co-twins. Supplemental Table 10 Differentially methylated CpG sites in myoblasts obtained from 14 subjects with type 2 diabetes compared with 14 unrelated controls among the 15,647 sites differentially methylated in muscle from the type 2 diabetes discordant twin pairs (*P* < 0.05). Supplemental Table 11 Differentially methylated CpG sites in myotubes obtained from 14 subjects with type 2 diabetes compared with 14 unrelated controls among the 15,647 sites differentially methylated in muscle from the type 2 diabetes discordant twin pairs (*P* < 0.05). Supplemental Table 12 Differentially expressed miRNAs in skeletal muscle from twins with type 2 diabetes compared with co-twins without type 2 diabetes (*P* < 0.05). miR family names from miRBase indicate the relatedness between the miRNAs. Families with more than one member are shown. Supplemental Table 13 Intra–twin-pair correlations between miRNA expression and 2-h glucose, fasting glucose levels and HbA1c (*P* < 0.05). Within–twin-pair differences of the measures were calculated by subtracting the value of the co-twin without type 2 diabetes from the value of the twin with type 2 diabetes and correlations were analyzed using Spearman statistics. Supplemental Table 14 This table presents 569 predicted targets of the 69 differentially expressed miRNAs presented in Supplementary Table 12 and which overlap with the 823 differentially expressed genes presented in Supplementary Table 5 using the multiMiR tool (http://multimir.ucdenver.edu/) which integrates the following tools: miRTarBase (https://mirtarbase.cuhk.edu.cn/~miRTarBase/miRTarBase_2022/php/index.php), TarBase (https://dianalab.e-ce.uth.gr/tarbasev9), DIANA-microT (https://dianalab.e-ce.uth.gr/microt_webserver/#/), MicroCosm (http://www.ebi.ac.uk/enright-srv/microcosm/), miRanda (http://www.microrna.org/), miRDB (https://mirdb.org/), PITA (http://mirtoolsgallery.tech/mirtoolsgallery/node/1066), ELMO (http://www.mirz.unibas.ch/), TargetScan (https://www.targetscan.org/vert_80/), PicTar (https://pictar.mdc-berlin.de/), and miRecords (https://www.hsls.pitt.edu/obrc/index.php?page=URL1237998207). Supplemental Table 15 Intra–twin-pair correlations between differences in miRNA expression and methylation in/near the gene (*P* < 0.05). Within–twin-pair differences of the measures were calculated by subtracting the value of the co-twin without type 2 diabetes from the value of the twin with type 2 diabetes and correlations were analyzed using Spearman statistics.

## Data Availability

The T2D discordant twins datasets generated for this study (DNA methylation data: accession number LUDC2023.11.2, mRNA data: accession number LUDC2023.11.3, and miRNA data: accession number LUDC2023.11.4) are deposited in the LUDC repository (https://www.ludc.lu.se/resources/repository). Data from the twins are available upon request and acceptance. Individual-level data from the twins are not publicly available due to ethical and legal restrictions related to the Swedish Biobanks in Medical Care Act, the Personal Data Act, and the European Union’s General Data Protection Regulation and Data Protection Act. mRNA expression and DNA methylation data from human myoblasts and myotubes, comparing individuals with T2D versus controls in either myoblasts or myotubes, are available in the NCBI Gene Expression Omnibus (GEO) with accession numbers GSE166467 (https://www.ncbi.nlm.nih.gov/geo/query/acc.cgi?acc=GSE166467) and GSE166652 (https://www.ncbi.nlm.nih.gov/geo/query/acc.cgi?acc=GSE166652), respectively. The dataset of skeletal muscle expression microarray samples from subjects with T2D or IGT and controls used for validation is publicly available at https://mae.crc.med.lu.se/mae2 (MuscleAtlasExplorer).

## Abbreviations

BCAA: Branched-chain amino acid
CpG: Cytosine guanine dinucleotide
ECM: Extracellular matrix
FBS: Fetal bovine serum
FDR: False discovery rate
GLUT4: Glucose transporter 4
GSEA: Gene set enrichment analysis
IGT: Impaired glucose tolerance
LNA: Locked nucleic acid
miRNA: MicroRNA
MZ: Monozygotic
NC: Negative control
NGT: Normal glucose tolerance
OGTT: Oral glucose tolerance test
RMA: Robust multichip average
RT: Reverse transcription
SNPs: Single nucleotide polymorphisms
T2D: Type 2 diabetes
