# Supplementary figures and images for: Multiomics profiling of DNA methylation, microRNA, and mRNA in skeletal muscle from monozygotic twin pairs discordant for type 2 diabetes identifies dysregulated genes controlling metabolism
Source: BMC Med. 2024 Dec 2;22:572. doi: 10.1186/s12916-024-03789-y (PMC11613913; doi:10.1186/s12916-024-03789-y)

A

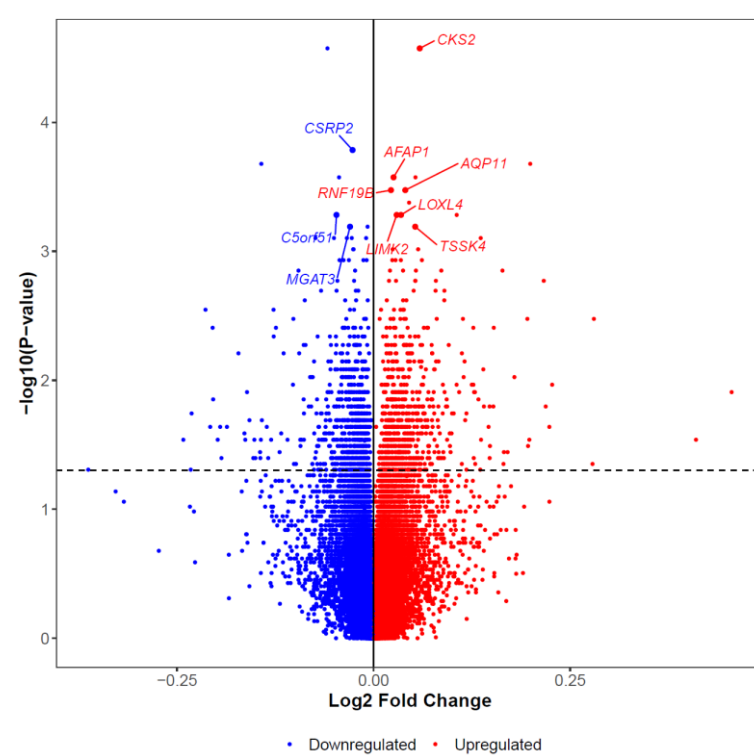

B

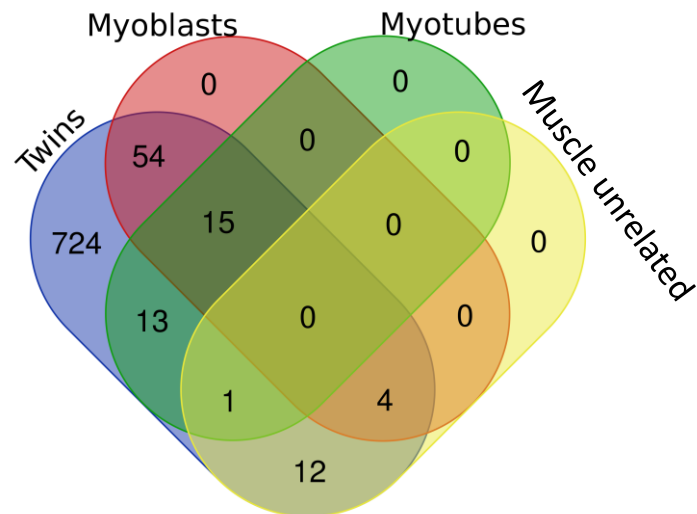

C

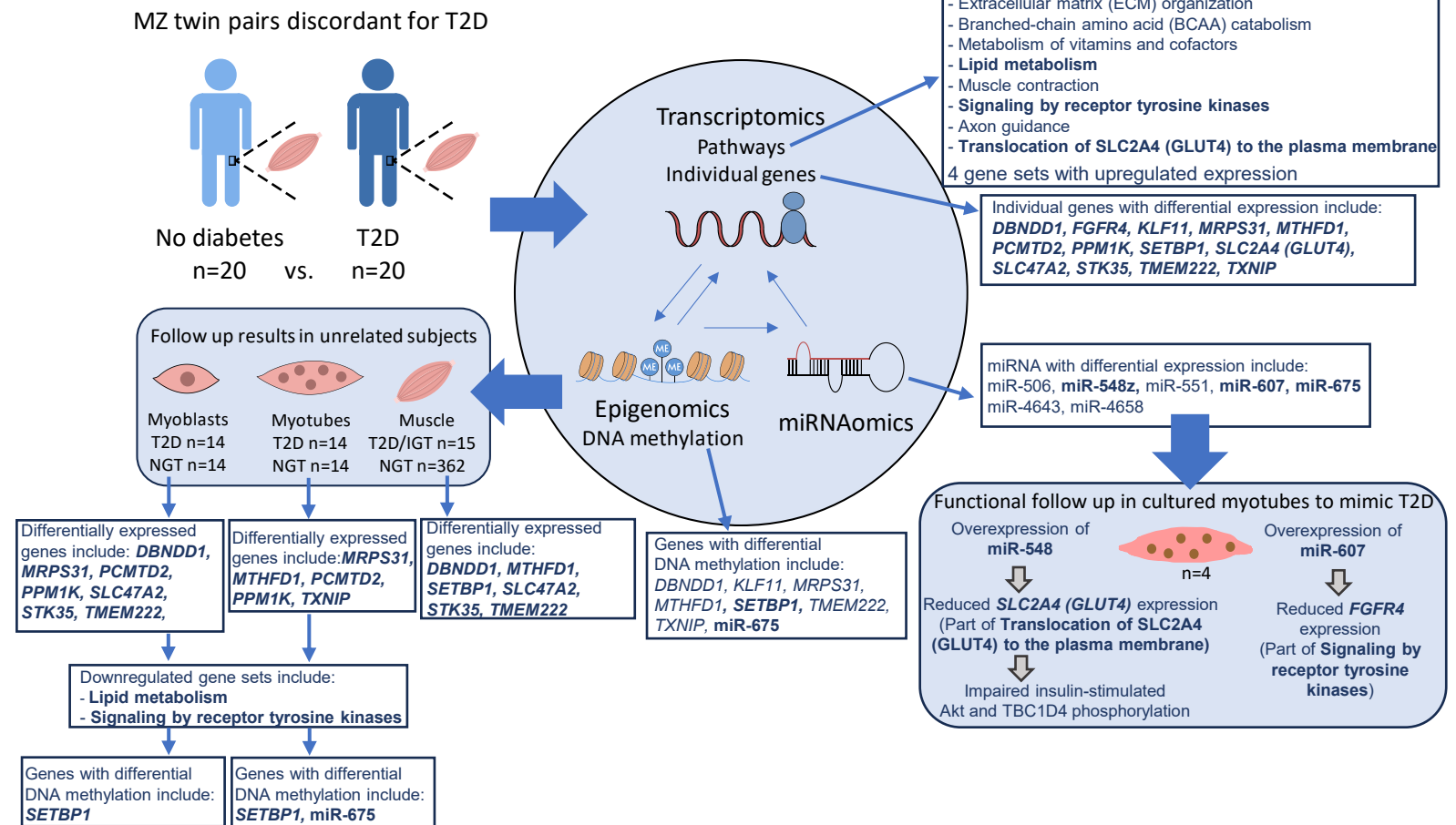

Supplement: Supplementary file 1 — Additional file 1: Supplemental Table 1 Clinical characteristics of donors of muscle cells. Supplemental Table 2 Genes contributing to the enrichment scores of significantly regulated pathways obtained from the gene set enrichment analysis in muscle tissue from twins with type 2 diabetes compared with co-twins without type 2 diabetes. Supplemental Table 3 Gene sets with differential expression in myoblasts from subjects with type 2 diabetes versus controls (gene set enrichment analysis with q < 0.05). Supplemental Table 4 Gene sets with differential expression in myotubes from subjects with type 2 diabetes versus controls (gene set enrichment analysis with q < 0.05). Supplemental Table 5 Differentially expressed genes between twins with type 2 diabetes and co-twins without diabetes (P < 0.05). Supplemental Fig. 1 (A) Differentially expressed genes between monozygotic (MZ) twins with type 2 diabetes (T2D) and co-twins without T2D based on nominal P values (P < 0.05). Log2 fold changes in the volcano plot are shown when comparing expression in muscle from MZ twins with versus co-twins without T2D. The dashed line indicates P < 0.05. Gene labels are based on the top 20 most significant transcripts, however of those, only 10 labels are displayed as there were no annotations for the other 10. (B) Overlap of genes with differential expression in the skeletal muscle from MZ twins discordant for T2D (P < 0.05), in skeletal muscle from 15 subjects with T2D or impaired glucose tolerance (IGT) versus 362 normal glucose tolerance (NGT) controls (q < 0.05), as well as in cultured myoblasts and myotubes from 13 T2D versus 13 NGT unrelated subjects (P < 0.05). These results are also presented in Supplemental Table 5. (C) Schematic summary, partly based on Fig. 1A, highlighting some key results from this study. Bold text marks findings, we could validate in muscle biopsies or cells from unrelated subjects and/or based on functional experiments overexpressing two miRNAs in cultured [file 12916_2024_3789_MOESM1_ESM.zip › Figure S1.pdf]
